# The effect of pelvic organ prolapse type on sexual function, muscle strength, and pelvic floor symptoms in women: A retrospective study

**DOI:** 10.4274/tjod.45722

**Published:** 2017-06-15

**Authors:** Nuriye Özengin, Hatice Çankaya, Elif Duygu, Muhammet Fatih Uysal, Yeşim Bakar

**Affiliations:** 1 Abant İzzet Baysal University, School of Kemal Demir Physical Therapy and Rehabilitation, Bolu, Turkey

**Keywords:** Pelvic organ prolapses, sexual function, muscle strength, pelvic floor symptoms

## Abstract

**Objective:**

This retrospective research was planned to investigate the effect of pelvic organ prolapse (POP) type on sexual function, muscle strength, and pelvic floor symptoms in symptomatic women.

**Materials and Methods:**

Data on POP type and stages as assessed using the Pelvic Organ Prolapse-Quantification system of 721 women who presented to the women’s health unit between 2009 and 2016 were collected retrospectively. POP types were recorded as asymptomatic, anterior, apical, and posterior compartment prolapses. Sexual function was assessed using the Pelvic Organ Prolapse/Urinary Incontinence Sexual Questionnaire short-form (PISQ-12), pelvic floor muscle strength was assessed through vaginal pressure measurement, and pelvic floor symptoms and quality of life were assessed using the Pelvic Floor Distress Inventory-20 (PFDI-20).

**Results:**

Among 168 women who met the inclusion criteria, 96 had anterior compartment prolapses, 20 had apical compartment prolapses, 16 had posterior compartment prolapses, and 36 women were asymptomatic. There was no difference between the groups in their PISQ-12 total and subscales scores, PFDI-20 total and two subscale (colorectal/anal, urinary) scores, and muscle strength (p>0.05). In the Pelvic Organ Prolapse Distress Inventory-6, another subscale of PFDI-20, it was determined that there was a difference between asymptomatic women and those with anterior compartment prolapses (p=0.044) and apical compartment prolapses (p=0.011).

**Conclusion:**

This research found that POP type did not affect sexual function, muscle strength, and colorectal and urinary symptoms in our cohort. There were more prolapse symptoms and complaints in women with anterior and apical compartment prolapses.

## PRECIS:

Pelvic organ prolapse type did not affect sexual function, muscle strength, and colorectal and urinary symptoms.

## INTRODUCTION

American College of Obstetricians and Gynecologists defined pelvic organ prolapses (POP) as prolapsing of the organs in the pelvis from inside and outside of the vaginal canal downward^(1)^. POP is described as anterior, posterior, and apical compartment prolapses according to their location in the vaginal canal^(2)^. POP symptoms may vary and may not necessarily be specific to any one compartment. Anterior compartment symptoms are urinary frequency, urgency, incontinence, intermittent flow, urinary difficulty, sense of incomplete discharge in the urinary bladder, and insufficient flow; posterior compartment symptoms are defecation difficulty, sense of incomplete discharge in bowels, constipation, and digital palpation need for discharge^(3,4)^. The only accepted symptom of POP seen in the three compartments is vaginal protrusion^(4)^. It was reported that POP prevalence ranged between 6-97% and POP affected 50% of women who gave birth to various degrees and 20% of these were asymptomatic^(4,5,6,7)^.

POP is a serious public health problem that affects sexual function, quality of life, and psychological state; however, it is generally ignored by women. It was reported in that POP affected sexual function and life quality of women negatively^(8,9,10,11)^. However, a limited number of studies have contributed data regarding how women are affected by POP in relation to the prolapse compartment^(12,13)^. The objective of this research was to investigate the effect of prolapse compartments on sexual function, muscle strength, pelvic floor symptoms, and quality of life in symptomatic women.

## MATERIALS AND METHODS

### Participants

This study was conducted retrospectively on 721 women who presented to Abant İzzet Baysal University, School of Kemal Demir Physical Therapy and Rehabilitation, Women’s Health Unit, between November 2009 and January 2016. The inclusion criteria for the research were determined as being clinically diagnosed with stage 1 and over POP, being aged more than 18 years, sexually active, and speaking Turkish. Women who had symptoms of urinary and fecal incontinence without POP, any mental problems that hindered comprehension, neurologic or psychiatric illness, pregnancy, pelvic surgery history, incomplete assessment form, and the same stage POP in more than one compartment were excluded. Approval from the institutional review board and informed consent form all the participants were obtained (approval number: 2014/17).

The women included in the research were divided into 4 groups; women with stage 2 and above POP symptoms: anterior, apical and posterior, and asymptomatic women with stage 1 prolapse. The physical features of the women [age, body height, body weight, body mass index (BMI)] and their sociodemographic information (educational status, profession, menstrual status, obstetric anamnesis, and medical history) were recorded. Pelvic Organ Prolapse-Quantification (POP-Q) assessment, pelvic floor muscle strength, Pelvic Organ Prolapse/Urinary Incontinence Sexual Questionnaire short-form (PISQ-12), and Pelvic Floor Distress Inventory-20 (PFDI-20) data were taken from the women’s files.

### Pelvic Organ Prolapse-Quantification

POP-Q assessments of the women were made by a physiotherapist specialized in urogynecologic physiotherapy. POP-Q is a quantitative standardized measurement method used for determining POP localization and level. After the women were placed in the lithotomy position, anterior (Aa and Ba), posterior (Ap and Bp), apical (C and D), total vaginal length (TVL), genital hiatus, and perineal body (PB) were measured using a rule and recorded in centimeters. In POP-Q assessment, with the exception for TVL, all values were recorded at maximal protrusion using the Valsalva maneuver. These measurement results were recorded in 3x3 table and POP staging was made. POP-Q stages range between 0 and 4, and a high stage indicates more serious prolapses(14). The women were classified as having anterior, apical, and posterior compartment POP, and asymptomatic POP based on these results.

### Pelvic floor muscle strength measurement

Pelvic floor muscle strength was assessed through vaginal pressure measurement (Myomed 932 Enraf/Nonius^®^). The women were positioned on their back with their hips and knees flexed. The contraction and resting periods of the device were adjusted to 10 seconds. After the placement of the vaginal probe into the vagina, the women were told to relax their pelvic floor muscles with the “relax” command, and to squeeze the placed vaginal probe and lift it inside without contracting their abdomen, hip and thigh muscles, and without holding their breath with the “contract” command. The measurement was repeated three times and pelvic floor muscle strength was recorded as hectopascal^(15)^.

### Pelvic Organ Prolapse/Urinary Incontinence Sexual Questionnaire-12

Sexual function of women who were sexually active during the past six months were assessed using the Turkish version of PISQ-12. PISQ-12 is a valid and reliable condition-specific questionnaire that assesses behavioral/emotive, physical, and partner-related aspects of sexual function. The questionnaire provides information about sexual desire and activity frequency and orgasmic characteristics. PISQ-12 assesses the effect of POP on sexual function, women’s perception, and how partners view pelvic floor disorders. In addition, it questions sexual function of partners. The questions are scored between 0 (never) and 4 (always), and questions 5-12 are estimated inversely. The maximum score obtainable from each section is 16. The total score achievable in the questionnaire ranges between 0-48. A high score indicates better sexual function^(16)^.

### Pelvic Floor Distress Inventory-20

Pelvic floor distress symptoms, quality of life, and pelvic floor dysfunction severity were assessed using the reliable and condition-specific Turkish version of PFDI-20. PFDI-20 consists of 3 subscales as Pelvic Organ Prolapse Distress Inventory-6 (POPDI-6), Urinary Distress Inventory-6 (UDI-6), and Colorectal-Anal Distress Inventory-8 (CRADI-8), and 20 questions. The subscale scores of PFDI-20 are 0-100 and the total scores (sum of three subscale scores) range from 0-300; high scores indicate more severe pelvic floor distress^(17)^.

### Statistical Analysis

Sample size requirement was calculated using G*Power Manual 3.1.2.9^(18)^. According to the outputs, a total of 112 women were required to ensure 80% power with an alpha level 0.05, and effect size was taken at the 0.4 level. The descriptive values of the obtained measurements were estimated as mean, median value, standard deviation, number and frequency. Whether the numerical characteristics showed normal distribution in each group was examined using the Shapiro-Wilk test. The Kruskal-Wallis test and post-hoc Dunn test were used for group comparisons. The statistical significance level was accepted as p≤0.05 and the SPSS version 20-demo program was used in the estimations.

## RESULTS

**Figure 1:**
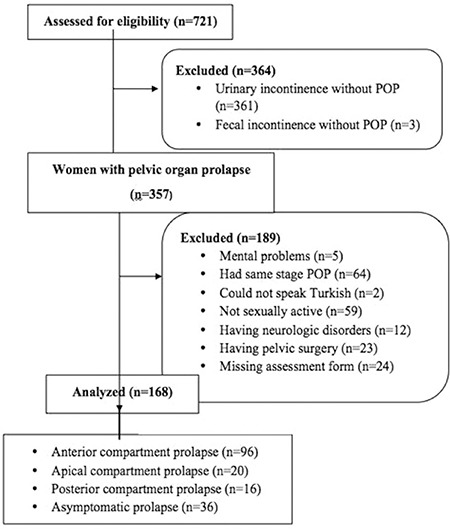
Enrollment diagram *POP: Pelvic organ prolapse*

Seven hundred twenty-one files were examined during this research. Three hundred sixty-one women among those were excluded for having urinary incontinence without POP, and 3 women were excluded for only having symptoms of fecal incontinence. When the remaining files were examined, it was determined that 357 women had POP. Among these, 5 women with mental problems, 64 women with the same stage POP in more than one compartment, 2 women who could not speak Turkish, 59 who had not had a sexual relationship within the past 6 months, 12 with neurologic disorders, 23 women with a history pelvic surgery, and 24 women whose assessment forms were incomplete were not included in the study. A total of 168 women, comprising 96 women with anterior compartment prolapse, 20 women with apical compartment prolapse, 16 women with posterior compartment prolapse, and 36 women with asymptomatic POP were included in the final analysis (Figure 1).

The physical and sociodemographic characteristics of the women included in the research are shown in Tables 1 and 2. There was no difference found in terms of age and height of asymptomatic women, women with anterior, posterior, and apical compartment prolapses (p>0.05). A statistical difference was determined in body weights (p=0.003) and BMI (p=0.011) of women with apical and anterior compartment prolapses.

There was no difference found between the groups in terms of PISQ-12 total scores and behavioral/emotive, physical, partner-related subscales, PFDI-20 total scores, and CRADI-8, UDI-6 subscales, and muscle strength (p>0.05, Table 3). POPDI-6 scores, a subscale of PFDI-20, exhibited statistically significant differences between asymptomatic women and women with anterior compartment prolapses (p=0.044) and apical compartment prolapses (p=0.011).

## DISCUSSION

This retrospective study, conducted to scrutinize the effect of POP type on sexual function of women, showed that different prolapse types did not affect sexual function. Sexual function has a critical role in women’s health, interest, and awareness in this field has been increasing gradually^(19)^. Sexuality depends on several factors including body image, socio-emotional perspective, sexual perception, quality of relationship with partner, and partner’s desire and competency^(20)^. It is known that POP mostly affects sexual function in the issues of sexual desire, orgasm ability, and arousal^(10,21,22)^. Psychological factors such as change in body image that could occur in women with POP, physiologic factors such as anatomic anomalies and diminished sensitivity in the genital region can lead to stimulation and orgasm disorders in women^(12,23)^.

Studies examining sexual function based on prolapse types are scarce. Mouritsen and Larsen^(13)^ showed that bladder, bowel, and sexual symptoms could be frequently seen in women with prolapses; however, they pointed out a very weak correlation between these symptoms and prolapse in a specific compartment. Lowenstein et al.^(12)^ reported that sexual function was not related with POP stage and vaginal compartment types but more related with body image perception and distress caused by POP level in women, rather than topographic changes based on POP. Lowenstein et al.^(12)^ concluded that women with poor body image had poor sexual functions. We found similar results in our research using the PISQ-12 to assess sexual function and by using the POP-Q system to determine prolapse type and stage. However, body image perceptions of these women were not able to be assessed due to the lack of a body image questionnaire specific to prolapse in the literature during the period when data were collected.

The behavioral/emotive subscale of sexual function did not alter according to POP types in this study. Nevertheless, the scores received in the behavioral/emotive subscale were lower than those of the physical and partner-related subscales. The behavioral/emotive subscale examines sexual desire, sexual relationship frequency, and orgasm state of women. It was interesting that the scores in this subscale in the 4 groups were lower when compared with the other subscales. According to the literature search, the averages of the behavioral/emotive subscales in other studies that assessed sexual function using PISQ-12 or PISQ questionnaires were higher than the averages of the women in our study^(22,24,25)^. The considerably low averages of the behavioral/emotive subscales in different POP types and the lack of a difference between the groups gave rise to the thought that this situation may not be related to POP but may be related to the conservative perspective of Turkish society towards sexuality. It is thought that factors such as sexual appetite is regarded as wrong in Muslim societies and sexuality among women is regarded as a requirement for having children and making their husbands happy, which causes repressed sexuality^(26)^.

It was previously reported that pelvic floor muscle strength, endurance, vaginal resting pressure, and pelvic floor muscle thickness decreased in women with POP^(27)^. To our knowledge, no studies have examined the effect of POP type on muscle strength. In this study, it was seen that pelvic floor muscle strength was similar between the groups. It was found that the sum of the muscle strength scores of the women with posterior compartment prolapse were higher than those of the apical and anterior compartment, and they were almost the same in asymptomatic women. This situation stems from the support of the posterior compartment by the PB and levator ani muscles, in addition to facial support^(28)^.

The PFDI-20 is used frequently for the assessment of pelvic floor symptoms of women^(2)^. In our study, it was found that the total scores of pelvic floor distress, urinary, and colorectal-anal symptoms were similar. Prolapse symptoms in women with anterior and apical compartment POP were found more severe when compared with asymptomatic women. To the best of our knowledge, there are no studies in the literature investigating pelvic floor distress symptoms according to POP types. We think that the reason for the more severe prolapse distress symptoms among women with anterior and apical compartment prolapses, and the absence of a statistical difference between women with posterior compartment prolapse and asymptomatic women was the support of posterior structures with strong power such as the levator ani. In addition, the unequal and widely distributed numbers of patients included in this retrospective study may have affected the results. It is known that increased body weight is a risk factor for POP^(29)^. We anticipated that the BMIs of asymptomatic women would have been lower in this study, but there was no difference between the compartments. It was observed that the BMIs of women with apical compartment prolapses were lower than those of women with anterior compartment prolapses.

The strength of this study was the inclusion of only women with POP, the same race of women, and using a condition-specific questionnaire for sexual function. When it is considered that muscle strength could also affect sexual function, objective and the quantitative measurement of pelvic floor muscle strength of women was another strength of this research. Despite the conservative perspective of Turkey towards sexuality, questioning sexual function of women in a rural province was an extraordinary situation for a Muslim society.

### Study Limitations

This research has a limitation due to its retrospective nature. It caused the exclusion of women who were sexually inactive because PISQ-12 is an appropriate questionnaire only for sexually active women. In 2013 the questionnaire was revised as the Pelvic Organ Prolapse/Incontinence Sexual Questionnaire, International Urogynecological Association-Revised (PISQ-IR), which also assesses sexually inactive women. However, the questionnaire could not be used because there was no Turkish version at the start date of the study. Another limitation of this study was the inclusion of only asymptomatic women with stage 1 prolapse as a control group and exclusion of women with stage 0 POP. Women with stage 0 POP were not accepted as the control group because they did not present to our women’s health unit due to the lack of symptoms. Despite the presentation of women with stage 0 POP to our unit with pelvic floor dysfunction symptoms, this situation was the reason for our exclusion criterion. The lack of questioning sexual intercourse frequency, vaginal dryness, and body image perception are among our other limitations. It was reported in the literature that body image perception affected sexual function in particular, rather than POP’s topographic changes^(12)^.

## CONCLUSION

This study illustrated that sexual function and muscle strength may not be affected by prolapse type. It was determined that there were more prolapse symptoms and complaints in women with anterior and apical compartment prolapses. When it is considered that POP can exist in more than one compartment simultaneously, more studies are needed including women with more than one compartment prolapse with larger samples. We think that investigating the effect of POP on sexual desire with the inclusion of sexually inactive women will be influential to determine the negative aspects caused by POP with the addition of PISQ-IR to the literature. Although there is no difference when comparing sexual function relative to compartments, low behavioral/emotive sub-domain scores of women may actually indicate problems with their orgasm, frequency of sexual intercourse, and sexual desire. For this reason, clinicians should examine women without discriminating compartments and women should be directed to appropriate treatment.

## Figures and Tables

**Table 1:**
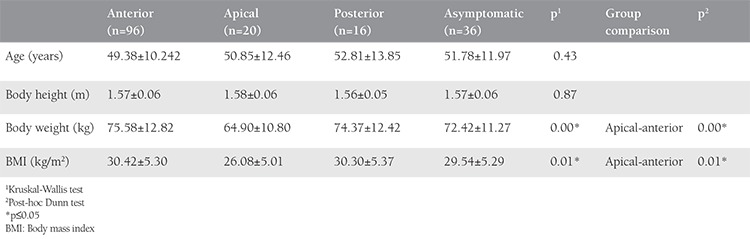
Physical features of the women by prolapse type

**Table 2:**
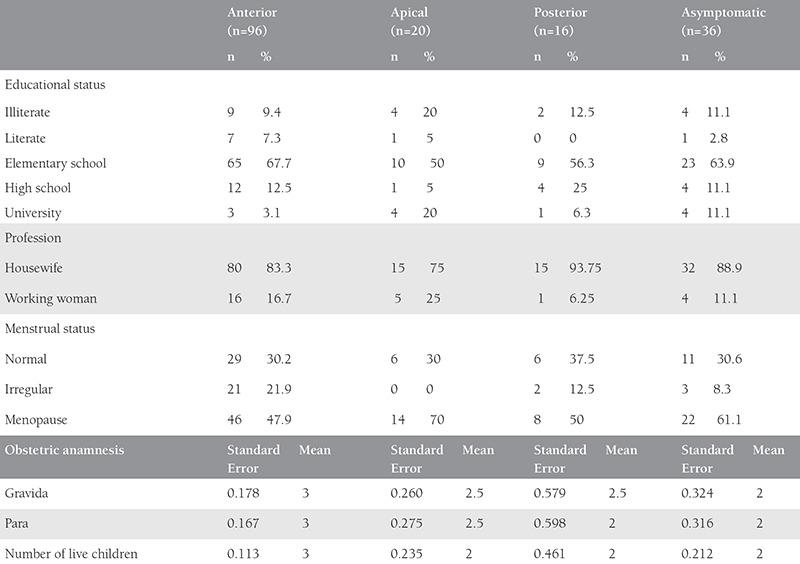
Sociodemographic characteristics of the women by prolapse type

**Table 3:**
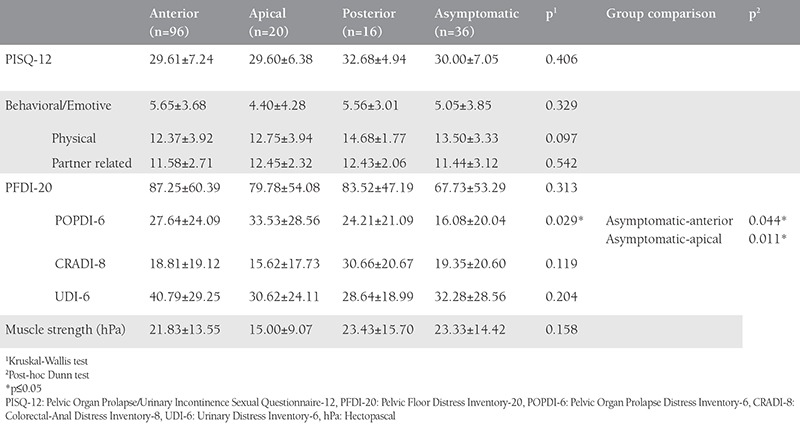
Pelvic Organ Prolapse/Urinary Incontinence Sexual Questionnaire-12, Pelvic Floor Distress Inventory-20 and muscle strength assessment by prolapse type
